# Health-related quality of life in patients with recurrent pericarditis: results from a phase 2 study of rilonacept

**DOI:** 10.1186/s12872-021-02008-3

**Published:** 2021-04-21

**Authors:** David Lin, Allan Klein, David Cella, Anna Beutler, Fang Fang, Matt Magestro, Paul Cremer, Martin M. LeWinter, Sushil Allen Luis, Antonio Abbate, Andrew Ertel, Leighann Litcher-Kelly, Brittany Klooster, John F. Paolini

**Affiliations:** 1 Abbott Northwestern’s Heart Hospital, Minneapolis Heart Institute, 800 East 28th Street, 2nd Floor, Minneapolis, MN 55407 USA; 2 Cleveland Clinic, Cleveland, OH USA; 3 Northwestern University, Evanston, IL USA; 4 Kiniksa Pharmaceuticals Corp., 100 Hayden Avenue, Lexington, MA 02421 USA; 5 University of Vermont Medical Center, Burlington, VT USA; 6 Mayo Clinic, Rochester, MN USA; 7 Virginia Commonwealth University, Richmond, VA USA; 8 Medstar Heart and Vascular Institute, Washington, DC USA; 9 Adelphi Values Patient-Centered Outcomes, Boston, MA USA

**Keywords:** Pericarditis, Interleukin-1 cytokine trap, Health-related quality of life, Recurrent pericarditis

## Abstract

**Background:**

Impact of recurrent pericarditis (RP) on patient health-related quality of life (HRQoL) was evaluated through qualitative patient interviews and as an exploratory endpoint in a Phase 2 trial evaluating the efficacy and safety of rilonacept (IL-1α/IL-1β cytokine trap) to treat RP.

**Methods:**

Qualitative interviews were conducted with ten adults with RP to understand symptoms and HRQoL impacts, and the 10-item Patient-Reported Outcomes Measurement Information System Global Health (PROMIS GH) v1.2 was evaluated to determine questionnaire coverage of patient experience. The Phase 2 trial enrolled participants with active symptomatic RP (A-RP, n = 16) and corticosteroid-dependent participants with no active recurrence at baseline (CSD-RP, n = 9). All participants received rilonacept weekly during a 6-week base treatment period (TP) plus an optional 18-week extension period (EP). Tapering of concomitant medications, including corticosteroids (CS), was permitted during EP. HRQoL was assessed using the PROMIS GH, and patient-reported pain and blood levels of c-reactive protein (CRP) were collected at Baseline and follow-up periods. A secondary, descriptive analysis of the Phase 2 trial efficacy results was completed using HRQoL measures to characterize both the impact of RP and the treatment effect of rilonacept.

**Results:**

Information from qualitative interviews demonstrated that PROMIS GH concepts are relevant to adults with RP. From the Phase 2 trial, both participant groups showed impacted HRQoL at Baseline (mean PROMIS Global Physical Health [GPH] and Global Mental Health [GMH], were lower than population norm average). In A-RP, GPH/MPH improved by end of base TP and were sustained through EP (similar trends were observed for pain and CRP). Similarly, in CSD-RP, GPH/MPH improved by end of TP and further improved during EP, during CS tapering or discontinuation, without disease recurrence (low pain scores and CRP levels continued during the TP and EP).

**Conclusion:**

This is the first study demonstrating impaired HRQoL in RP. Rilonacept treatment was associated with HRQoL improvements using PROMIS GH scores. Maintained/improved HRQoL during tapering/withdrawal of CS without recurrence suggests that rilonacept may provide an alternative to CS.

*Trial registration*: ClinicalTrials.Gov; NCT03980522; 5 June 2019, retrospectively registered; https://clinicaltrials.gov/ct2/show/NCT03980522.

## Introduction

Pericarditis, or inflammation of the pericardium, has a variety of etiologies but is most commonly referred to as “idiopathic” [1, 2]. The primary symptom of pericarditis is debilitating chest pain. Pericarditis is considered recurrent if symptoms and inflammation recur at least 4 weeks after an initial acute episode [1]. Recurrent pericarditis (RP) affects approximately 15–30% of patients who have an acute episode of pericarditis, and up to 50% of patients who experience one recurrence will experience two or more [1]. Empiric “off-label” therapy with nonsteroidal anti-inflammatory drugs [NSAIDs] and colchicine is often used successfully to treat the first pericarditis episode or initial recurrence [3]. Treatment options for patients with multiple recurrences, however, are limited, and there is a high unmet medical need for patients who have inadequate response (i.e., continued recurrence or incomplete symptom resolution) to, who cannot tolerate, standard therapy [1] or who have persistent underlying disease. Given that the cytokines interleukin-1 alpha (IL-1α) and beta (IL-1β) are implicated in RP etiology [2, 4], rilonacept, an IL-1 α and IL-1 β cytokine trap, was evaluated in clinical trials for the treatment of RP [5, 6] and is now the first treatment approved by FDA for RP.

While the impact of RP on patients’ health-related quality of life (HRQoL) has been reported in the literature [7–12] and is thought to be due to the primary symptom of the condition (e.g., chest pain) and the resulting uncertainty and anxiety about new recurrences, impact on HRQoL has not been explicitly evaluated in previous clinical research. In addition, corticosteroids (CS), despite well-known warnings and precautions in patients with cardio-metabolic comorbidities, are widely used to treat RP [13], putting patients at risk for additional adverse events, including recurrence and steroid dependence [14]; comorbidities associated with chronic CS use may also lead to adverse impacts on HRQoL [15, 16].

Both qualitative and quantitative research approaches were used to explore the HRQoL impacts experienced by adults with RP and how those impacts may change in response to treatment. Qualitative interviews were conducted with ten adults with RP to document the patient experience of RP symptoms and HRQoL impacts (known as concept elicitation interviews). Results from these interviews were used to develop a conceptual model of RP. In addition, a Phase 2 clinical trial of rilonacept for the treatment of RP included an HRQoL patient-reported outcome (PRO) questionnaire as an exploratory endpoint. Therefore, the objective of these two streams of research is to evaluate HRQoL in patients with RP: specifically, to confirm whether the concepts assessed with the HRQoL PRO questionnaire used in the Phase 2 clinical trial are relevant to patients with RP (based on patient reports during the qualitative interviews) and to evaluate the effect of rilonacept treatment on physical and mental aspects of HRQoL.

## Methods

The sections below describe the methodology used for both the qualitative interviews with patients and the clinical trial study design relevant to the current research objective.

### Methods for qualitative patient interviews to develop a patient-centric conceptual model

To understand the patient experience of RP, one-on-one telephone interviews were conducted with adults with a confirmed diagnosis of RP.

#### Participants for qualitative interviews

The qualitative interview study was approved by a centralized independent review board (IRB); following approval, potentially eligible participants were identified from clinical sites through review of medical records. Key inclusion criteria for the qualitative study included: age 18 years or older and a clinical diagnosis of RP (either idiopathic or due to post-pericardiotomy syndrome, adult onset Still’s Disease, or Dressler’s Syndrome), defined as the first episode of acute pericarditis (as defined by the 2015 European Society of Cardiology Guidelines for the Diagnosis and Management of Pericardial Diseases) [1] followed by at least one pericarditis recurrence after a symptom-free interval of at least 4–6 weeks. Key exclusion criteria for the study included: individual was currently enrolled in another clinical interventional study for RP; individual had a diagnosis of RP that was secondary to specific prohibited etiologies, including tuberculosis, neoplastic, purulent, or radiation, post-thoracic blunt trauma, myocarditis, or systemic autoimmune diseases (with the exception of adult onset Still’s Disease).

Potentially eligible participants were presented with study information by a recruiting clinician (or his/her representative), and once participants provided a signed informed consent form, the clinical site completed a screening document to determine participants’ eligibility. Participant interviews were scheduled once eligibility was confirmed.

#### Interview conduct

One-on-one, 60-min telephone interviews with ten adults aged 18–75 years with RP were conducted. Interviewers used a semi-structured interview guide to facilitate a conversational-style interview and included open-ended questions to understand the patient experience of RP and its treatments, specifically what signs, symptoms, and HRQoL impacts are experienced in relation to RP from the patient perspective.

Interview guide questions included:

- “Could you please start by telling me about the first signs or symptoms of [participant’s term for RP] you noticed?
- “Does the [patient-reported sign/symptom] have any impact on your daily life? If so, how?”
- “Have there been any changes to your daily life because of [participant’s term for RP]? If so, can you please describe?”

#### Data handing and analysis

Interviews were audio-recorded after obtaining participant consent, transcribed, and anonymized. These transcripts were coded and qualitatively analyzed using the ATLAS.ti software program (ATLAS.ti Scientific Software Development GmbH, Berlin, Germany). The goal of transcript coding was to organize and catalog participants’ descriptions of the characteristics of RP, in order to develop a patient-centric conceptual model of RP signs, symptoms, and impact concepts. A conceptual model is a heuristic classification scheme that links a specified disease state or condition to its proximal and increasingly distal health outcomes [17], acts as a framework for understanding a disease and/or its treatment, specifies the potentially relevant outcomes for a program of research, and informs the selection of measurement concepts to foster the development of questionnaires, outcomes, and endpoints. Specifically, to characterize the specific applicability of the Patient Reported Outcome Measurement Information System Global Health (PROMIS GH v1.2) [17] questionnaire (described below) for capturing the HRQoL impacts experienced by patients with RP, conceptual mapping was conducted to compare the concepts within the conceptual model against PROMIS GH v1.2 individual items.

### Methods for the Phase 2 study KPL-914-C001

The methodology of the Phase 2 clinical trial of rilonacept for the treatment of RP (clinicaltrial.gov: NCT03980522) is provided in detail by Klein and colleagues [5]. To summarize, this was a multicenter, open-label, single-active-arm Phase 2 study that enrolled two specific patient populations of adults with RP: (1) patients with an active recurrence who were symptomatic and had signs of inflammation at baseline (A-RP) and (2) patients who were not having an active recurrence but were dependent on CS (CSD-RP). All participants received weekly subcutaneous (SC) injections of rilonacept for 6 weeks during the treatment period (TP) and were invited to continue weekly SC injections for up to 18 weeks in the extension period (EP).

### Participants

Adults (18–75 years of age) with RP (idiopathic or post-pericardiotomy syndrome etiology) were enrolled and stratified into one of two participant groups, A-RP and CSD-RP. Participants in the A-RP group either (1) had evidence of elevated c-reactive protein (CRP) at baseline or (2) did not have elevated CRP, potentially due to concomitant medications (such as CS) but had evidence of pericardial inflammation by cardiac magnetic resonance imaging. Participants in the CSD-RP group had CS-dependent disease (based on information from the investigator regarding prior recurrences when taking medication) and did not have active pericarditis symptomatology or elevated CRP at baseline.

#### Assessments

Blood levels of CRP were evaluated weekly in TP, and then monthly, to measure inflammation from baseline to the end of the EP for all participants. In addition, two PRO questionnaires were completed by all participants during the clinical trial. They included:

1. A single-item 11-point numeric rating scale (NRS) for average pericarditis pain intensity with a 24-h recall window (with 0 = no pain to 10 = pain as bad as it could be) [18–20] was completed weekly in TP and monthly in EP from baseline to the end of the EP.
2. The 10-item PROMIS GH v1.2 questionnaire was also completed by participants at up to five timepoints during the clinical trial to assess HRQoL [21]. The analyses presented here focused on the following three most critical timepoints: baseline (Day 0), end of TP (Week 6), and Final Visit (end of EP). Items 1–7 ask participants to think about their general health and are rated on a five-point response scale (with higher scores associated with better quality of life). Items 8–10 ask participants to report on the emotional problems, fatigue, and pain over the last seven days, with Items 8 and 9 rated on a five-point response scale (with higher scores associated with better quality of life) and Item 10 rated on a 0–10 NRS (with higher scores associated with more pain). Two domain scores are created from the 10-item scale, the global physical health (GPH) and the global mental health (GMH). The GPH is scored by averaging together the global03 (physical health), global06 (physical function), global07 (pain) and global08 (fatigue) items. The GMH is scored by averaging together the global02 (quality of life), global04 (mental health), global05 (satisfaction with discretionary social activities), and global10 (emotional problems) [21]. The published US-generalized normative scores for both of these domains are a mean score of 50 and a standard deviation (SD) of 10 [21].

#### Procedure and analyses

All participants received rilonacept SC injections weekly for 6 weeks until the end of TP and were invited to continue weekly SC injections (at the same dose) during an optional 18-week EP. For those on other concomitant medications for RP at baseline (participants in both the A-RP and CSD-RP groups), including CS, the option to taper was offered during the EP. Participants completed the PRO questionnaires (PROMIS and Pain NRS) at study visits, including telephone and site visits. Blood levels for CRP were also assessed during clinical site visits or via visiting nurse or local contract laboratory; CRP was analysed via a central laboratory.

The analyses presented in the results are descriptive, given the small sample size of the clinical trial and the single-active-arm design. Specifically, results are reported as means and SD, with ranges of values for each participant group. While participants were asked to complete the HRQoL questionnaire at multiple timepoints in the clinical trial, the analyses focus on the baseline, (Day 0), end of base TP (Week 6), and Final Visit at end of EP timepoints. Effect sizes (ESs; Cohen’s d) were calculated with 95% confidence intervals (CI) to evaluate the magnitude of the change from baseline to end of EP for each patient group for the HRQoL scores. ESs ≥ 0.80 were considered large; ≥ 0.50 to < 0.80, medium; and ≥ 0.2 to < 0.5, small [22]. In addition, the descriptive analyses also include the weekly pericardial pain NRS scores and CRP blood levels that were collected at study visits.

## Results

### Qualitative patient interviews

Qualitative interviews were conducted via telephone with ten adults diagnosed with RP to understand the patient experience of the condition, including the signs, symptoms, and HRQoL impacts. Participants were recruited from three clinical sites in the US. The mean age of the participants was 58.5 years (SD = 11.5), and six participants (60.0%) were female. Clinicians reported that participants exhibited the following RP types: idiopathic (n = 4, 40.0%), post-pericardiotomy syndrome (n = 4, 40.0%), adult-onset Still’s Disease (n = 1, 10.0%), and Dressler’s syndrome (n = 1, 10.0%). The majority of participants reported taking over-the-counter or prescription anti-inflammatory medications (n = 7, 70.0%), and half reported that they had previously taken CS (n = 5, 50.0%) for their RP.

**Fig. 1:**
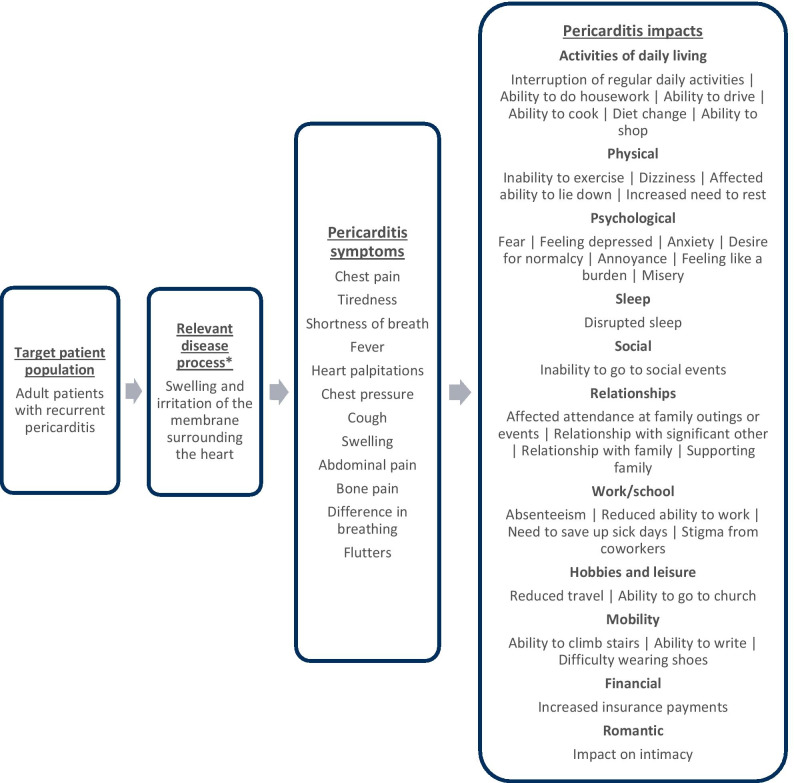
Patient-centric conceptual model for recurrent pericarditis. Proposed by Wilson and Cleary [17], a conceptual model is a heuristic classification scheme that links a specified disease state or condition to its proximal and increasingly distal health outcomes. This model presents the concepts reported by adult RP patients during qualitative interviews: RP symptoms (proximal to the disease process of RP) and impacts to daily life (organized by HRQoL domain, increasingly distal to the disease process). *Khandaker et al. [26]

A total of 13 symptoms and 34 impacts across 11 domains were reported by participants during these qualitative interviews and are summarized in Table 1 and were organized into a conceptual model (Fig. 1). All participants reported experiencing chest pain (n = 10, 100.0%), with seven (n = 7, 70.0%) stating it is the most bothersome symptom, and five (n = 5, 50.0%) reporting it is the most important symptom to improve. After chest pain, the next most frequently reported signs or symptoms (reported by at least half of the participants) were tiredness (n = 8, 80.0%), shortness of breath (n = 7, 70.0%), fever (n = 6, 60.0%), and heart palpitations (n = 5, 50.0%). The most frequently reported impacts (reported by at least half of the participants) were inability to exercise (n = 8, 80.0%), disrupted sleep (n = 7, 70.0%), fear (n = 6, 60.0%), inability to go to social events (n = 6, 60.0%), interruption of daily activities (n = 6, 60.0%), absenteeism (n = 5, 50.0%), and impaired ability to do housework (n = 5, 50.0%).

**Table 1:** Patient-reported recurrent pericarditis symptom and impact domains description table

Symptom or impact domain reported by participant	Description^a^	Frequency of participant reports^b^
		(N = 10)
		n (%)
*Symptoms*		
Chest pain	Described as sharp, stabbing, dull, or aching pain or pressure in the chest, which can radiate to the neck and shoulders	10 (100.0%)
Tiredness	Described as physical exhaustion lasting a few days, which may co-occur with shortness of breath and affect one’s activity level	8 (80.0%)
Shortness of breath	Described as difficulty breathing and losing breath quickly, similar to a feeling of suffocation	7 (70.0%)
Fever	Described as a low-grade fever that can include hot flashes or chills	6 (60.0%)
Heart palpitations	Described as the heart beating rapidly and arrhythmically and causing discomfort	5 (50.0%)
Chest pressure	Described as discomfort or heaviness in the chest	3 (30.0%)
Cough	Described as uncomfortable and painful sporadic coughing episodes triggered by a tickling feeling	3 (30.0%)
Swelling	Described as swollen feet and legs that feel tight and bloated, possibly associated with lack of circulation	2 (20.0%)
Abdominal pain	Described as intense pain above the navel	1 (10.0%)
Bone pain	Described as bearable pain in the bones, feeling like soreness in the upper back	1 (10.0%)
Difference in breathing	Described as difficulty breathing, both as difficulty inhaling deeply and breathing deeper and longer than usual	1 (10.0%)
Flutters	Described as an uncomfortable sensation of the heart beating rapidly	1 (10.0%)
Neck pain	Described as sharp nerve pain in the neck affecting neck mobility	1 (10.0%)
*Impact domains*		
Activities of daily living	Described in the following ways *Inability to complete plans and daily activities* *Inability to begin or complete household tasks, such as cleaning, cooking, and/or yard work* *Impacts on driving (or fear of driving due to symptoms)* *Diet and lifestyle changes* *Inability to go shopping*	9 (90.0%)
Physical impacts	*Described in the following ways*	8 (80.0%)
	*Difficulty exercising and restrictions on exercising*	
	*Feeling dizzy (due to shortness of breath)*	
	*Inability to lay down (due to pain and shortness of breath)*	
	*Feeling the need to rest (due to heart palpitations)*	
Psychological impacts	*Described in the following ways* *Feeling scared because of symptoms* *Feeling depressed because of symptoms* *Feeling anxious, worried, or concerned because of chest pain* *Not feeling normal and wanting to feel normal (due to symptoms)* *Annoyance due to symptoms* *Feeling like a burden to others* *Feeling miserable due to chest pain*	8 (80.0%)
Sleep impacts	*Described in the following ways* *Waking up frequently or suddenly after falling asleep (associated with chest pain and shortness of breath)*	7 (70.0%)
Social impacts	*Described in the following ways* *Not being able to go out with friends or attend events*	6 (60.0%)
Relationship impacts	*Described in the following ways* *Not being able to go to family events* *Emotional distance or less activity with significant other* *Feeling distant from family* *Not being able to support family as much*	6 (60.0%)
Work or school impacts	*Described in the following ways* *Not being able to go to work* *Not being able to work as much or as effectively* *Saving up sick hour to take off when experiencing symptoms* *Feeling less comfortable with coworker*s	6 (60.0%)
Hobbies or leisure impacts	*Described in the following ways* *Inability to travel or go on vacations* *Inability to attend church*	4 (40.0%)
Mobility impacts	*Described in the following ways* *Impact on climbing stairs* *Difficulty writing due to chest pain experienced into the shoulder* *Shoes feeling uncomfortably tight due to swelling of feet*	3 (30.0%)
Financial impacts	*Described in the following ways* *Increased co-pays due to the condition*	1 (10.0%)
Romantic impacts	*Described in the following ways* *Not feeling intimate with significant other as a result of symptoms*	1 (10.0%)

^a^Description of concept summarized based on reports by study participants

^b^Frequency is presented as the total number and percentage of all study participants who reported each concept

In order to confirm that the assessment of HRQoL completed by participants in the Phase 2 clinical trial captured concepts relevant to adults with RP, concepts reported during the qualitative interviews were mapped to the ten items of the PROMIS GH v1.2 questionnaire. Table 2 shows the results of this exercise, with representative patient quotes from the qualitative interviews for each of the items of the PROMIS GH questionnaire. In particular, adults reported symptoms and HRQoL impacts during the interviews that are included in the PROMIS GH questionnaire, such as pain, social and emotional impacts, and physical functioning.

**Table 2:** Conceptual mapping of the PROMIS GH v1.2 questionnaire to the qualitative results from patient interviews

PROMIS GH Items	Reported by patients during qualitative interviews	Representative patient quotes from N = 10 qualitative interviews^a^
Item 1: In general, would you say your health is	✓	[INTERVIEWER]: … has this heart inflammation caused any psychological impacts or impacted the way you feel?
		[PATIENT]: I know it sounds crazy to say, but sometimes I just want to be normal. But what is normal?.… Because I think it's more of a hindrance. Like, I feel like my body's falling apart
Item 2: In general, would you say your quality of life is	✓	[INTERVIEWER]: … has anything changed since … you first visited the doctor for those symptoms [sharp chest pain and trouble breathing]?
		[PATIENT]: Uh, my quality of life has.… I would do, like, walks. Um, I would walk three miles a day. And now I – sometimes I can't even get started when, um, when I have the symptoms
Item 3: In general, how would you rate your physical health?	✓	[INTERVIEWER]: Are there any other impacts that you experience to your life?
		[PATIENT]: Um, I used to be able to ride a bike.… And that I can't do as much
Item 4: In general, how would you rate your mental health, including your mood and your ability to think?	✓	[INTERVIEWER]: … how bad would you say it is [patient described symptoms as ‘sharp stabbing in the chest’] … if you had to describe, you know, how severe or bad it is?
		[PATIENT]: I mean, I'm pretty much just not the same person.… I guess I'm just a miserable person. Even my wife tells me I'm a miserable person to be around when – when I'm going through these episodes
Item 5: In general, how would you rate your satisfaction with your social activities and relationships?	✓	[INTERVIEWER]: Is there anything else that you kind of changed, or, um, stopped doing, or do less now, because of the pericarditis?
		[PATIENT]: I used to go out, you know, with friends, stay out late at night. That I don't do anymore
		[INTERVIEWER]: … would you say that it [recurrent pericarditis] affects your ability to engage in social or leisure in your life?
		[PATIENT]: … it has stopped me from socializing with family gatherings. You know, I just – I'm too tired to even get ready and go out and do a 3-day weekend
Item 6: In general, please rate how well you carry out your usual social activities and roles. (This includes activities at home, at work and in your community, and responsibilities as a parent, child, spouse, employee, friend, etc.)	✓	[INTERVIEWER]: are there specific examples that you're thinking of that, when you feel tired, you can't do as well?
		[PATIENT]: I just can't finish [mopping and sweeping]. Like, I'll do half the house, and then I'll just have to take – sit down
		[INTERVIEWER]: is there any other impact it [recurrent pericarditis] has on your daily life?
		[PATIENT]: I mean I can't be active with my grandkids
Item 7: To what extent are you able to carry out your everyday physical activities such as walking, climbing stairs, carrying groceries, or moving a chair?	✓	[INTERVIEWER]: How severe would you say that [tiredness feeling] is, in general?
		[PATIENT]: I still kind of tried to work out, because I always tried to be as healthy as possible. And it – it was just too hard for me to do
		[INTERVIEWER]: Can you describe what that [fatigue or tiredness] feels like?
		[PATIENT]: Um, exhaustion.… I just I love to walk, well, that's eliminated. I don't do much walking. I'm just so tired
Item 8: How often have you been bother by emotional problems such as feeling anxious, depressed, or irritable?	✓	[INTERVIEWER]: You mentioned fatigue. Um, can you go into a little bit more detail about that, how that feels – you know, how it affects your day?
		[PATIENT]: Well, when you have the acute episode, you know, you're going through all that pain, and all the other symptoms – palpitations and shortness of breath, and maybe a little low grade fever, and uh, that cough's coming off and on, the stabbing pains – all that is very taxing on your system, you know. And it's really denting. It's depressing to have all these symptoms. And you're fearful, you're extremely fearful
		[INTERVIEWER]: …what did the shortness of breath feel like?
		[PATIENT]: It's frightening, it's scary. You think you're going to smother. And – and you think you're going to die. It's like you're – you're underwater, and you can't get oxygen
Item 9: How would you rate your fatigue on average?	✓	[INTERVIEWER]: which one would you say is the most bothersome symptom and why?
		[PATIENT]: Hmm, the most that bothers me – I would say palpitations. But actually, I don't get those as often as how I'm feeling fatigue. Do you know what I mean? I've gotten to the point where I'm so fatigued that I – I really can't get out of bed
Item 10: How would you rate your pain on average?	✓	[INTERVIEWER]: I just wanted to talk to you a bit about your … the sharp, stabbing chest pain you talked about before.… I was just hoping you could describe it a little bit more
		[PATIENT]: I would say sharp pain more like, um, like if, like if a elephant's sitting on me so I'm suffocating
		[INTERVIEWER]: I'd like to talk a little bit more in detail about, um, each of these things that you've mentioned … so, you said the chest pain, um, sort of feels like a stabbing?
		[PATIENT]: Yes, stabbing pain.… Like needles

^a^Qualitative interviews were conversational in nature, and patient quotes determined to be representative of PROMIS GH measurement concepts resulted from various lines of questioning

PROMIS GH = Patient Reported Outcome Measurement Information System Global Health

### Phase 2 study rilonacept

Twenty-five participants were enrolled in a multicenter, open-label, single-active-arm Phase 2 clinical trial of rilonacept, with an average age of 42.8 ± 10.5 years (± indicates SD; range 26–62); most were female (n = 15, 60.0%) and white (n = 22, 88.0%). Participants had a mean number of prior recurrences of 2.6 (range 1–8), average duration of disease of 2.2 ± 1.9 years (range 0.2–7.9 years), and average number of pericarditis episodes per year of 3.9 ± 3.7 (range 0.54–15). Based on their baseline symptoms and signs of pericardial inflammation, there were two groups of participants: those experiencing an active recurrence who were symptomatic with evidence of inflammation (A-RP; n = 16), and those who were CS-dependent but not acutely symptomatic at baseline (CSD-RP; n = 9). See Table 3 for the demographics and health characteristics of these two participant groups.

**Table 3:** Demographics and health characteristics of Phase 2 clinical trial sample

Characteristic	Active recurrence (A-RP)	Not symptomatic, Corticosteroid-dependent (CSD-RP)
	N = 16	N = 9
Age (years) (Mean ± SD [range])	39.8 ± 10.52 (26–58)	48.2 ± 8.56 (36–62)
Gender (% female [n])	75.0% (n = 12)	33.3% (n = 3)
Race (% white [n])	81.3% (n = 13)	100% (n = 9)
BMI (kg/m^2^) (Mean ± SD [range])	31.99 ± 7.51 (23.4–52.7)	28.97 ± 4.68 (22.5–34.3)
Duration of disease (years) (Mean ± SD [range])	2.6 ± 2.13 (0.2–7.9)	1.4 ± 0.97 (0.6–3.4)
Number of prior recurrences (median, [range])	2 (1–8)	3 (2–5)
Baseline NRS Pain Rating 0–10 (Mean ± SD [range])	4.6 ± 1.82 (2–8)	1.4 ± 1.51 (0–5)
Baseline CRP values (mg/dL) (Mean ± SD [range])	3.8 ± 5.30 (0.09–19.84)	0.19 ± 0.11 (0.05–0.36)
*Concomitant medications at baseline*		
Aspirin (n [%])	0 (0%)	2 (22.2%)
NSAID (n [%])	7 (43.8%)	5 (55.6%)
Colchicine (n [%])	12 (75.0%)	8 (88.9%)
CS (n [%])	6 (37.5%)^a^	9 (100.0%)^b^

BMI = body mass index; CRP = c-reactive protein; CS = corticosteroids; EP = extension period; NRS = numeric rating scale; NSAID = nonsteroidal anti-inflammatory drug; SD = standard deviation

^a^4/6 (66.7%) discontinued CS and 1/6 (16.7%) tapered CS by end of EP; 1/6 (16.7%) did not enter EP

^b^7/9 (77.8%) discontinued CS and 1/9 (11.1%) tapered CS by end of EP; 1/9 (11.1%) did not enter EP

**Fig. 2:**
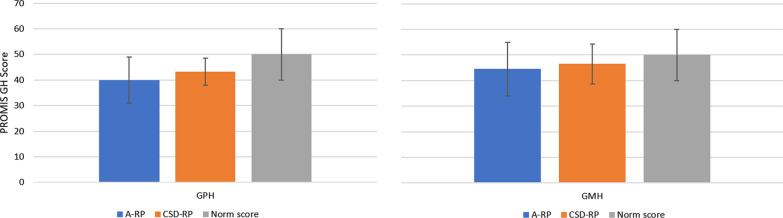
Mean PROMIS GPH/MPH at Baseline for A-RP and CSD-RP. This figure presents the mean and standard deviations for the baseline scores of the PROMIS GH physical (GPH) and mental (GMH) health domains. For both the A-RP (n = 16) and CSD-RP (n = 9) groups, average scores for these domains are below the US normative average score of 50. A-RP = active symptomatic recurrent pericarditis; CSD-RP = corticosteroid-dependent recurrent pericarditis with no active recurrence; GPH = Global Physical Health; GMH = Global Mental Health; PROMIS GH = Patient-Reported Outcomes Measurement Information System Global Physical Health

Scores from the PROMIS GH questionnaire items and domains were evaluated for each of the participant groups over time (baseline, end of TP, and end of EP). Figure 2 shows the baseline scores for the two domains of the PROMIS GH health questionnaire. For both the A-RP and CSD-RP groups, average scores for these domains are below the US normative average score of 50. Additionally, Table 4 shows a trend for improvement in some item and domain scores for both the A-RP and CSD-RP groups.

**Table 4:** PROMIS GH item and domain scores over time (mean ± SD), by participant group

PROMIS GH item/ domain^a^	Active recurrence (A-RP)				Not symptomatic, Corticosteroid-dependent (CSD-RP)			
	Baseline (n = 16)	End of TP visit (n = 15)	End of EP visit (n = 15)	ES (95%CI)^b^	Baseline (n = 7)	End of TP visit (n = 9)	End of EP visit (n = 8)	ES (95%CI)^b^
GPH	39.94 ± 8.94	51.35 ± 7.96	51.32 ± 6.56	**1.44** (0.65 to 2.23)	43.30 ± 5.31	45.09 ± 4.06	46.81 ± 9.27	0.46 (− 0.57 to 1.48)
Item 3: physical health	2.6 ± 0.96	3.2 ± 1.01	3.5 ± 0.83	**1.00** (0.25 to 1.75)	2.8 ± 0.46	3.1 ± 0.33	3.0 ± 0.93	0.27 (− 0.75 to 1.29)
Item 7: physical activities	3.3 ± 1.39	4.4 ± 1.06	4.1 ± 1.03	0.65 (− 0.07 to 1.37)	3.4 ± 0.74	3.3 ± 0.87	3.8 ± 1.04	0.44 (− 0.59 to 1.46)
Item 9: fatigue	3.1 ± 0.96	3.7 ± 0.49	3.7 ± 0.82	0.67 (− 0.05 to 1.39)	3.1 ± 0.69	3.2 ± 0.44	3.4 ± 1.06	0.33 (− 0.69 to 1.35)
Item 10: pain	4.8 ± 1.88	0.6 ± 1.18	0.5 ± 1.13	**-2.69** (− 3.66 to − 1.72)	1.7 ± 1.60	1.0 ± 1.32	1.4 ± 2.50	-0.14 (− 1.16 to 0.87)
GMH	44.50 ± 10.48	50.13 ± 11.33	50.54 ± 11**.**00	0.56 (− 0.16 to 1.28)	46.49 ± 7.77	47.91 ± 5.51	50.66 ± 6.30	0.59 (− 0.44 to 1.63)
Item 2: quality of life	3.0 ± 1.03	3.6 ± 1.06	4.0 ± 1.00	**0.98** (0.24 to 1.73)	3.3 ± 1.04	3.6 ± 0.73	3.4 ± 0.74	0.11 (− 0.90 to 1.13)
Item 4: mental health	3.3 ± 1.13	3.7 ± 1.23	3.6 ± 1.12	0.27 (− 0.44 to 0.97)	3.4 ± 0.74	3.6 ± 0.73	3.9 ± 0.83	0.63 (− 0.41 to 1.67)
Item 5: social activities and relation-ships	3.1 ± 1.34	3.7 ± 1.18	3.6 ± 1.12	0.40 (− 0.31 to 1.12)	3.1 ± 0.83	3.3 ± 0.50	3.6 ± 0.92	0.57 (− 0.47 to 1.60)
Item 8: emotional problems	3.1 ± 1.41	3.5 ± 1.36	3.4 ± 1.12	0.23 (− 0.47 to 0.94)	3.4 ± 0.98	3.3 ± 0.71	4.0 ± 0.53	0.78 (− 0.27 to 1.83)
*Items that are not included in above domains*								
Item 1: general health	2.9 ± 0.72	3.5 ± 0.83	3.6 ± 0.91	**0.82** (0.12 to 1.59)	2.9 ± 0.64	3.1 ± 0.33	3.1 ± 0.64	0.31 (− 0.71 to 1.33)
Item 6: social activities and roles	3.1 ± 1.09	3.5 ± 1.25	3.5 ± 1.13	0.36 (− 0.35 to 1.07)	2.9 ± 0.99	3.1 ± 0.93	3.5 ± 0.93	0.63 (− 0.41 to 1.67)

CI = confidence interval; EP = extension period; ES = effect size; GMH = Global Mental Health; GPH = Global Physical Health; PROMIS GH = Patient Reported Outcome Measurement Information System Global Health; TP = treatment period

^**a**^For Items 1–9, scores range from 1 to 5 with higher scores indicating improvement, and for Item 10, scores range from 0 to 10 with lower scores indicating improvement. Scoring for Item 10 is adjusted when calculating the GPH. To calculate the GMH and GPH domain scores, raw scores are converted to standardized T scores, with a normative mean of 50 and standard deviation of 10

^b^ES is calculated from Baseline and End of EP Visit; Bolded values are large (≥ 0.80)

For participants in the A-RP group, increases in the average scores for items of the PROMIS GH questionnaire that assess general health, quality of life, and physical health indicate improvement over the study period, with large ESs. In addition, for the A-RP group, the average score of the PROMIS GH pain item shows the largest decrease over the study period, with a mean score of nearly 5 on the 0–10 NRS at baseline, and less than 1 at end of EP (ES = − 2.69; 95%CI = − 3.66 to − 1.72). At baseline, both the physical and mental domain scores (GPH/GMH) were lower than the normative average of 50, but by end of TP mean scores for the GPH were above the US norm (and remained above at end of EP, with a large change [ES = 1.48; 95%CI = 0.65 to 2.23), and mean scores for the GMH were at the US norm (and remained at the normative average at end of EP).

For participants in the CSD-RP group, there were modest increases (improvements) on the PROMIS GH items assessing mental health, social activities and relationships, social activities and roles, and emotional problems, with ESs indicative of medium changes (Cohen’s d between 0.5 and 0.8). The average scores for the other items did not change. Similar to the A-RP group, average scores for the CSD-RP group were also below the US norm for both the GPH and GMH domains at baseline. For the GMH domain, average scores were at the normative average at the end of EP.

**Fig. 3:**
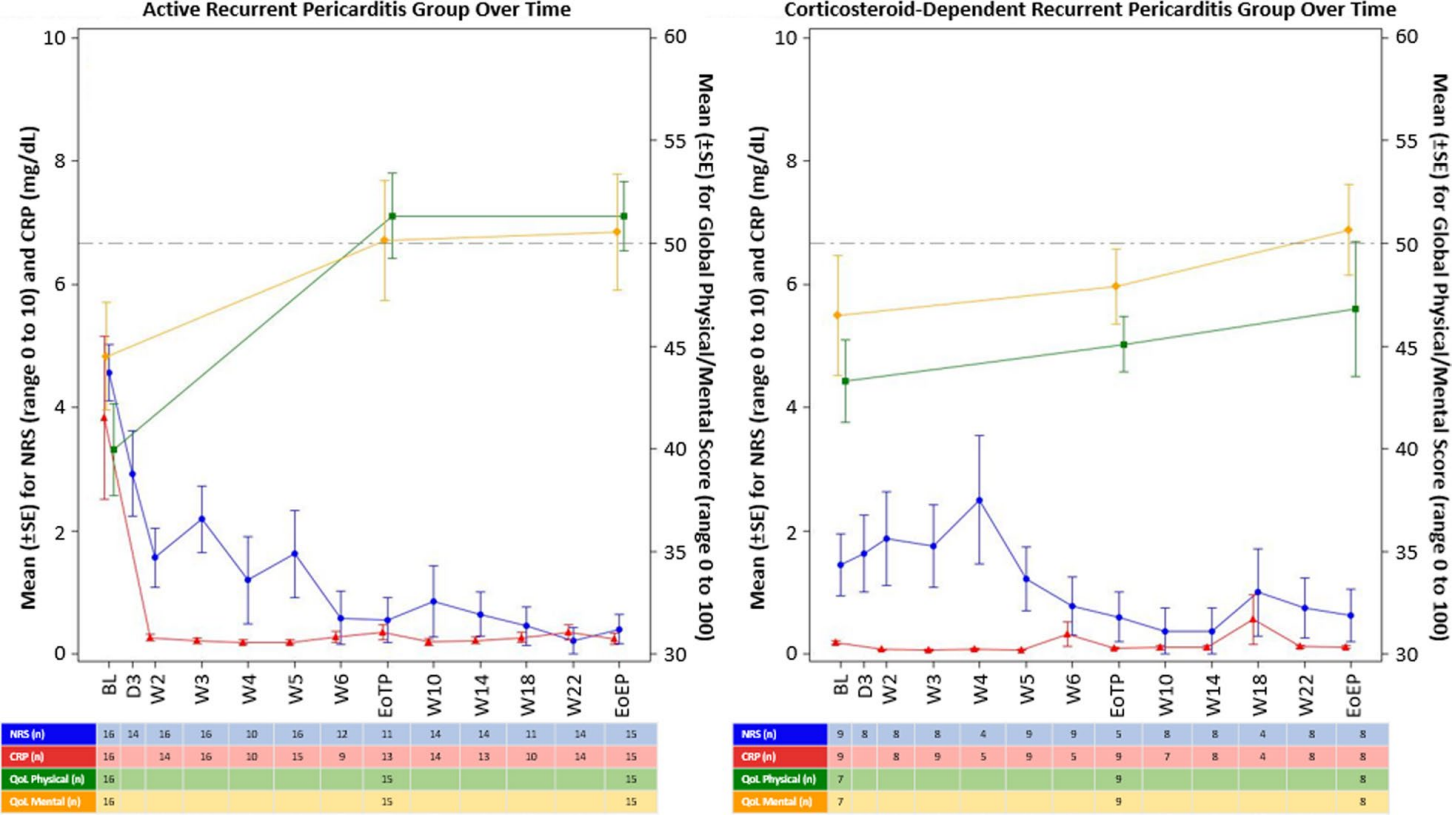
PROMIS GH domain scores, pericardial pain, and c-reactive protein levels over time by participant group. This figure shows the change in the GPH and GMH domain scores over the study period for the A-RP group and the CSD-RP group, and the trend between these HRQoL scores and patient-reported pericardial pain and serum marker of inflammation (CRP). For the A-RP group, pain scores and CRP levels decrease over the study period, while HRQoL scores increase. For the CSD-RP group, pericardial pain and CRP (low at baseline, as expected because these participants entered the trial while not in active flare) remain low over the course of the study even while tapering and discontinuing CS, while HRQoL scores increase over time. BL = baseline; CRP = c-reactive protein; D = day; EoEP = end of extension period; EoTP = end of treatment period; NRS = numeric rating scale; QoL = quality of life; SE = standard error; W = week

Figure 3 shows the change in the GPH and GMH domain scores over the study period, along with the trend between these HRQoL scores and patient-reported pericardial pain and serum marker of inflammation (CRP). For the A-RP group, pain scores and CRP levels decreased over the study period (change in pain scores from Baseline to end of EP was 4.6 ± 1.82 to 0.4 ± 0.91 [ES = − 2.89, 95%CI = − 3.90 to − 1.88]; change in CRP from Baseline to end of EP was 3.84 ± 5.30 to 0.24 ± 0.36 [ES = − 0.94, 95%CI = − 1.89 to − 0.20]), while HRQoL scores increased. For the CSD-RP group, pericardial pain and CRP (low at baseline, as expected as these participants entered the trial while not in active recurrence) remained low over the course of the study even while tapering and discontinuing CS (change in pain scores from Baseline to end of EP was 1.4 ± 1.51 to 0.6 ± 1.19 [ES = − 0.58; 95%CI = − 1.56 to 0.39]; change in CRP from Baseline to end of EP was 0.19 ± 0.11 to 0.12 ± 0.06 [ES = − 0.78; 95%CI = − 1.76 to 0.21]), while HRQoL scores increase over time.

Please note that participants who completed the EP and were taking CS at Baseline (all participants in the CSD-RP [n = 8], and 83.3% [n = 5/6] of participants in the A-RP) were able to taper and/or discontinue using CS by the end of the EP (i.e., the end of the study) without recurrence or pericarditis symptomatology (e.g., patient-reported pericardial pain) or inflammation (e.g., elevated CRP level).

## Discussion

While a substantial negative impact of RP on patients’ HRQoL has traditionally been assumed, to our knowledge this is the first analysis using qualitative and quantitative methods to explore the ways that symptoms of pericarditis recurrence impact patients’ quality of life. The results from the baseline timepoint of the Phase 2 clinical trial align with the assumption of patients’ decreased HRQoL, showing that scores on the GMH and GPH of the PROMIS GH questionnaire were on average lower than normative scores for both the A-RP and CSD-RP groups. In addition, the improvement in HRQoL scores over the course of the study tracks with improvements in patient-reported pericardial pain and CRP levels for the A-RP group, and with the tapering and discontinuing of CS for the CSD-RP group while pericardial pain and CRP remained stable and low while on rilonacept treatment. For the A-RP group, the magnitude of change between Baseline and End of EP was large for the PROMIS GH physical health, pain, and quality of life items, and the GPH domain (Cohen’s d ≥ 0.80). In contrast, for the CSD-RP group, the changes between Baseline and End of EP for the mental health item and the GMH domain (Cohen’s d 0.50 to < 0.80), while not large (which was expected, given the absence of acute pericarditis recurrences) were not insubstantial, likely due to the tapering and discontinuation of corticosteroids. Future studies should investigator further the impact of CS use on HRQoL, and how discontinuation of CS for patients with RP on targeted therapy impacts physical and emotional HRQoL. Taken together, these results show that RP negatively impacts patients’ quality of life physically and emotionally, and that improvements in quality of life may be associated with improvement in disease symptomatology and a decrease in pericardial inflammation, in particular, while patients receive targeted treatment.

Furthermore, results from qualitative interviews, where adults with RP spoke about the unpredictable nature of the condition, supported that pericarditis recurrences impact patient physical and mental health. Specifically, using the qualitative data, a conceptual model of RP was developed, and HRQoL concepts included in the concept model (e.g., ability to carry out daily activities, impacts on mood, and limitation on social activities) were mapped against the ten items of the PROMIS GH v1.2 questionnaire (included in the Phase 2 clinical trial of rilonacept), which demonstrate that this questionnaire is assessing concepts that are relevant to adults with RP.

Limitations include the small sample sizes for both the qualitative interviews and the clinical trial, the single-active-arm design of the clinical trial, and the relatively short duration (24 weeks) of the clinical trial compared to the overall duration of this chronic disease. In addition, while the inclusion criteria for the qualitative interview study were intended to be similar to those of the clinical trial, they were less restrictive (i.e., adults interviewed did not experience as many recurrences as the participants in the clinical trial). Nevertheless, these results provide preliminary support for the importance of including a multidimensional assessment of HRQoL for future clinical research of RP. It is also important to consider that some HRQoL impacts may be dependent on age and gender, therefore, given the age range of the participants who completed the qualitative interviews and the Phase 2 clinical trial, the resulting conceptual model should be considered representative of adult RP.

Strengths include leveraging qualitative results to support the importance of the item- and domain-level scores of the PROMIS GH v1.2 questionnaire to adults with RP. The representative patient quotes help contextualize how participants may be interpreting each item of the PROMIS GH questionnaire. In addition, the means for the PROMIS GH domain scores at baseline in the rilonacept clinical trial provide evidence of the impact RP has on patients’ HRQoL, as they are lower compared to population norm scores. These findings are consistent with other clinical studies reporting lower PROMIS GH questionnaire scores and associated impacts in physical, mental, and social domains in cardiac and vascular populations [23–25]. The increase in both the GPH and GMH scores over the course of the study for both the A-RP and CSD-RP, in conjunction with improvements and/or stable pericardial pain scores and CRP levels, shows that HRQoL scores may also be responsive to treatment as the patient’s condition improves, particularly when on a treatment that addresses IL-1 driven pericardial inflammation.

## Conclusions

Given the anxiety associated with the unpredictability of recurrences and the exercise restrictions that patients are expected to adhere to following a diagnosis of RP, it is important to evaluate both emotional and physical impacts of the condition. As more clinical trials move to incorporate patient-centric outcomes to evaluate treatments not only in terms of resolution of a physiological indicator of disease but also to ensure that patients feel and function better, future clinical trials of adults with RP should include HRQoL PRO questionnaires. In addition, future studies should explicitly examine the effect of concomitant medications, including CS, and their independent impact on patient HRQoL.

The results of this pilot study may suggest a signal of a positive impact of rilonacept on clinical outcome measures and improvements in patient HRQoL over the study time period which tracked with improvements in pericardial pain and inflammation. For those participants in the CSD-RP group, who were weaning off CS while taking rilonacept, patient-reported pericardial pain and CRP levels were stable, while HRQoL scores improved over the course of the study, without recurrences. With the approval of rilonacept, appropriate patients with RP may now have a CS-sparing treatment alternative that not only reduces pain and inflammation but also reduces the risk of recurrence while improving HRQoL.

## Data Availability

Data will be made available upon reasonable request.

## Abbreviations

A-RP: Active recurrence of pericarditis at baseline
CRP: C-reactive protein
CS: Corticosteroids
CSD-RP: Dependent on corticosteroids at baseline
EP: Extension period
GMH: Global mental health
GPH: Global physical health
HRQoL: Health-related quality of life
IRB: Independent review board
NRS: Numeric rating scale
NSAIDs: Nonsteroidal anti-inflammatory drugs
PRO: Patient-reported outcome
PROMIS GH: Patient Reported Outcome Measurement Information System Global Health
RP: Recurrent pericarditis
SC: Subcutaneous
TP: Treatment period
